# CAT: A Compound Attachment Tool for the Construction of Composite Chemical Compounds

**DOI:** 10.1021/acs.jcim.2c00690

**Published:** 2022-10-31

**Authors:** Bas van Beek, Juliette Zito, Lucas Visscher, Ivan Infante

**Affiliations:** # Division of Theoretical Chemistry, Faculty of Science, Vrije Universiteit Amsterdam, de Boelelaan 1083, Amsterdam 1081 HV, the Netherlands; β Dipartimento di Chimica e Chimica Industriale, Università degli Studi di Genova, Via Dodecaneso 31, Genova 16146, Italy; † Department of Nanochemistry, Istituto Italiano di Tecnologia, Via Morego 30, Genova 16163, Italy; ‡ BCMaterials, Basque Center for Materials, Applications, and Nanostructures, UPV/EHU Science Park, Leioa 48940, Spain; ¶ Ikerbasque Basque Foundation for Science Bilbao 48009, Spain

## Abstract

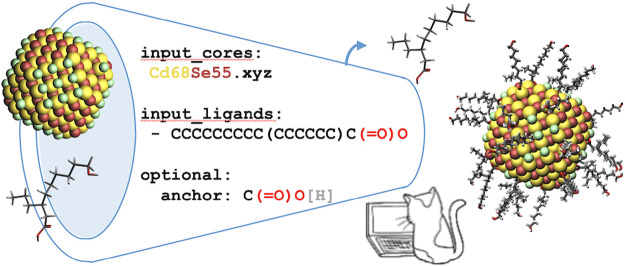

The continuous improvement of computer architectures allows for the simulation of molecular systems of growing sizes. However, such calculations still require the input of initial structures, which are also becoming increasingly complex. In this work, we present CAT, a Compound Attachment Tool (source code available at https://github.com/nlesc-nano/CAT) and Python package for the automatic construction of composite chemical compounds, which supports the functionalization of organic, inorganic, and hybrid organic–inorganic materials. The CAT workflow consists in defining the anchoring sites on the reference material, usually a large molecular system denoted as a scaffold, and on the molecular species that are attached to it, *i.e.*, the ligands. Usually, ligands are pre-optimized in a conformation biased toward more linear structures to minimize interligand(s) steric interactions, a bias that is important when multiple ligands are attached onto the scaffold. The resulting superstructure(s) are then stored in various formats that can be used afterward in quantum chemical calculations or classical force field-based simulations.

## 1 Introduction

Advances in computer hardware architectures are opening new routes to allow simulations of large composite chemical systems. Meanwhile, recent breakthroughs in the application of machine learning algorithms in computational chemistry, for example, for the prediction of chemico-physical properties of chemical structures, require efficient preparation and execution of thousands of calculations to generate a sufficient amount of training data.^1−4^ For all such atomistic simulations, a recurring task is the generation of initial structures consisting of Cartesian coordinates of all atoms in the system. Without these structures, no calculation is possible, and in many cases, this step forms a critical bottleneck in the workflow of designing and running the simulation on a supercomputer.^5,6^ In particular, the functionalization with organic molecules of nano- or bulk materials, whether they are organic, inorganic, or mixed organic–inorganic, can turn in a complex task because structures are more varied and less predictable by low-level theories. In these cases, there is a pressing need for fast and simple tools to aid in structure generation.

Usually, building initial structures for the nano- or bulk materials to be functionalized, also named as scaffolds from now on, is nontrivial; however, there are already tools available to tackle their preparation. In the case of purely inorganic scaffolds, their (crystal) structure can be generated using software tools like the pymatgen libraries^7^ and the Atomic Simulation Environment (ASE),^8^ which are able to build 2D and 3D structures of arbitrary ion combinations. These periodic structures, as well as nonperiodic cuts of a number of unit cells, can be translated into input formats for a range of quantum chemistry codes such as VASP,^9−11^ Quantum Espresso,^12,13^ ADF,^14^ Gaussian,^15^*etc*. Computational results produced on the basis of these structures can subsequently be stored in databases such as the Materials Project,^16^ NOMAD,^17^ AiiDA,^18^ or ioChem-BD^19^ to allow for data mining and machine learning applications. Nowadays, all these databases offer facilities to quickly browse the properties of a plethora of inorganic materials. In the case of organic scaffolds, such as dyes,^20^ proteins,^21,22^ and catalysts,^23^ there are three common ways to generate atomistic structures. The simplest is to manually construct the desired structure with the aid of a graphical user interface (*e.g.*, ADF-GUI,^14^ Molden,^24^ Avogadro,^25^*etc*.). This method works well when a few medium-sized molecules made of dozens of atoms need to be studied and has the advantage that the researcher has full control over the generated structures. The second method is to first (automatically) extract molecular structures from available databases such as PubChem,^26^ GDB-13,^27^ or QM7.^28^ These databases typically only provide molecules in SMILES^29,30^ or SELFIES^31^ formats meaning that these molecules still need to be converted into three-dimensional structural models with the aid of cheminformatics tools such as RDKit^32^ or OpenBabel.^33^ The third approach is to not select a particular molecule beforehand but instead design one with desired properties *via* generative adversarial networks,^34^ a machine learning-based architecture nowadays mostly employed for drug discovery.^35−37^ Finally, in the case of hybrid (organic–inorganic) material scaffolds, we rely either on building the desired structures manually using a graphical user interface or by mining available datasets built on purpose for a specific set of materials. A well-known example of hybrid materials with a large chemical space is represented by metal–organic frameworks (MOFs), where the growing variety of 3D porous MOFs and MOF-like frameworks^38,39^ has endeavored the creation of constantly updated machine-readable databases^40,41^ to store the available crystal structures.^42^ Another class of mixed materials is represented by hybrid perovskite materials of ABX_3_ composition, characterized by an inorganic network of corner-sharing BX_6_ octahedra (where B is a metal and X is a halogen) with organic A cations occupying the voids in between.^43^ Typical examples include CH_3_NH_3_PbI_3_ and CH(NH_2_)_2_SnI_3_, which employ respectively the methylammonium and formamidinium as A cations.

In most cases, if not all, the scaffolds described above are functionalized by a plethora of organic ligands introducing additional challenges, since the combination of organic molecules and inorganic materials leads to an explosion in the number of possible combinations and relevant structures that need to be built. Examples of functionalized materials include inorganic surfaces terminated with organic surfactants such as in self-assembled monolayers (SAMs)^44^ and colloidal nanoparticles.^45,46^ The latter represents a wide class of novel materials with promising optoelectronic and/or catalytic properties.^47,48^ Examples are nanocrystal semiconductors consisting of group II-VI, III-V, and IV-VI elements (examples being HgTe, InP, and PbS, respectively),^49−51^ metal halide perovskites,^52^ and nanoparticles consisting of purely metallic systems, such as gold, silver, and platinum.^53−55^ A key role played by the ligands here is stabilizing the inorganic scaffolds in organic solvents, preventing their dissolution.^56^ The suitability of a certain ligand for this purpose depends on factors such as interligand packing, magnitude of the scaffold/ligand binding strength, and ligand/solvent interactions.^57−59^ These criteria, augmented by practical considerations such as the ease of synthesis and material costs, in principle define an optimization target for ligand passivation. In the experimental practice, however, only a limited set of ligands is currently employed on a regular basis. For example, in colloidal nanocrystals, oleic acid and oleylamine are the most known passivating agents due their availability and low cost. Nevertheless, there remains a large unexplored space of potentially interesting ligands available within datasets such as Pubchem,^26^ GDB-13,^27^ and QM7.^28^

As the vast chemical space prohibits high-throughput experimental screening, an (initial) computational screening provides an attractive alternative. To facilitate such screening and allow for the exploration of many ligand-passivated scaffolds, it is desirable to have a tool that can automate the preparation of starting structures for these complex composite systems. While a number of such tools have recently emerged, *e.g.*, to obtain molecular models of organometallic species,^60−63^ no counterparts are currently available for more generalized scaffolds. We thus have developed a Python library called CAT (Compound Attachment Tool), which is intended for the construction of composite chemical compounds resulting from the functionalization of organic, inorganic, or mixed organic–inorganic materials with organic molecules. CAT has already been introduced, in an early prototype form, for the automatic generation of new dyes’ structures obtained by addition of one or more ligands (alcohols, amines, thiophenes, *etc.*) to an organic scaffold (1,4,5,8-naphthalenediimide).^64^ To disclose the full potential and general applicability of our tool, we decided to apply it on ligand-passivated colloidal nanocrystals and metal–organic frameworks, *i.e.*, on the functionalization of composite scaffolds. To facilitate the construction of all these composite chemical compounds, CAT possesses several features that we will discuss in more detail below. Features include, among others, automatic ligand functional group recognition, partial surface passivation of the scaffold, and minimization of interligand steric interactions *via* a biased conformational search.

## 2 Results and Discussion

### 2.1 CAT Workflow

**Figure 1:**
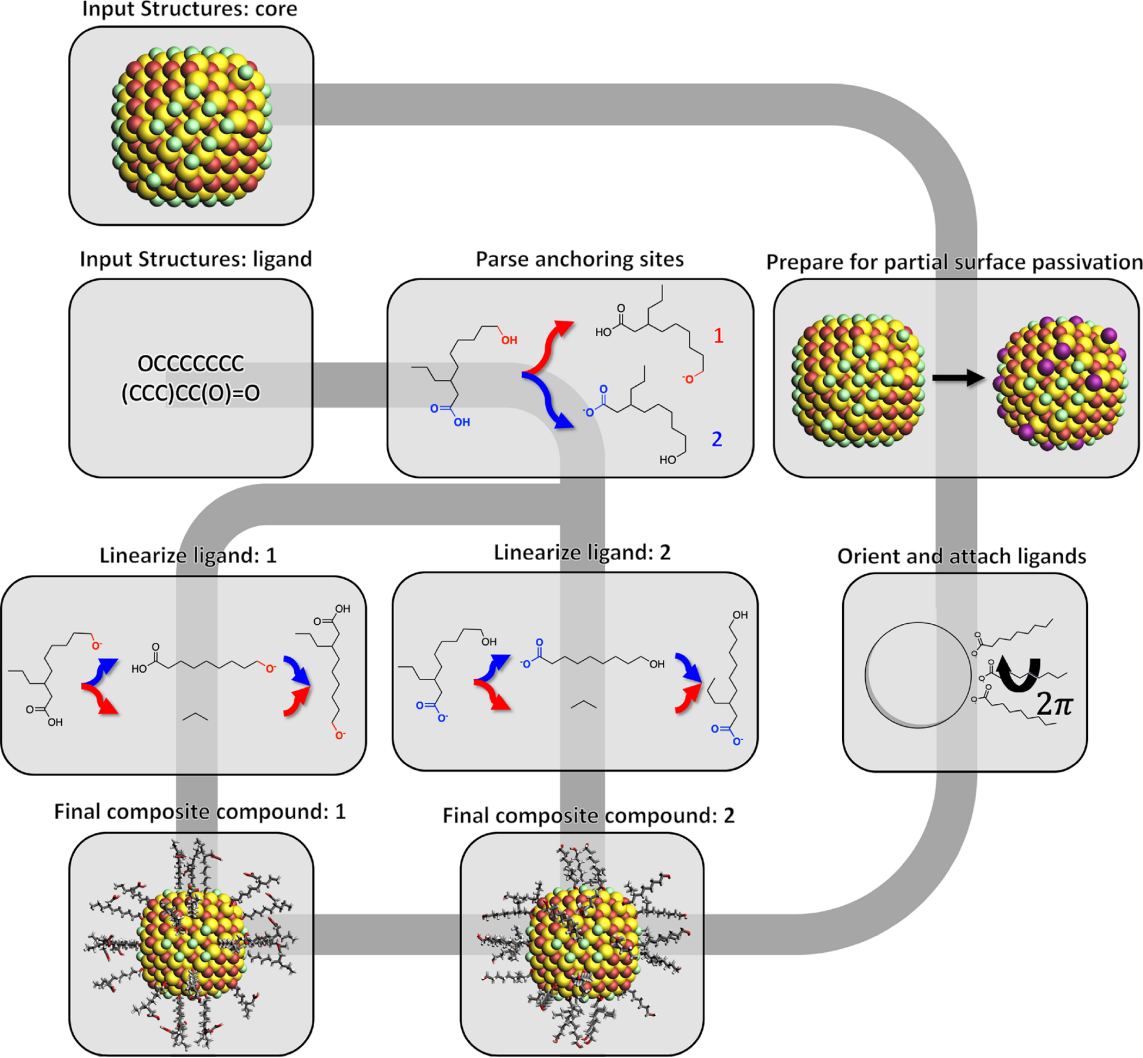
Schematic overview of the general CAT workflow.

The user of CAT should provide information about the type of structure that is to be generated, which includes the scaffold and the ligands that are to be attached to it. These user-specified settings are provided *via* a YAML file^65^ (Figure S1), a human-readable format aimed at data serialization. The two most important options to be provided are the input_cores and input_ligands keywords, the former representing a common moiety or scaffold (*e.g.* a CdSe nanocrystal) that will be functionalized with the latter (*e.g.* a carboxylic acid or its carboxylate base conjugate) at specific anchoring points. The dummy atoms serving as anchoring sites should generally be provided by the user. If unspecified, CAT will either default to chlorine for the scaffold or a wide range of common functional groups for the ligand, including, among others, hydroxides, amines, and phosphines. In the model used for illustration, the core dummy atoms are the chlorides, indicated by a green color (Figure 1). If multiple cores and/or ligands are provided, all possible combinations between a single scaffold and a ligand will be automatically constructed.

As we have constructed our software on top of the PLAMS^66^ library for automating molecular simulations, we can import structures in a variety of formats. These formats include, for instance, SMILES strings,^29,30^ Protein Databank format (PDB), XYZ, *etc.*(67) Regardless of the input, the structures are first converted to PLAMS Molecule objects, which are then internally used by CAT for representing the various chemical compounds. For the organic ligands, there is the advantage of being able to work with compact string notation schemes such as SYBYL,^68^ InChI,^69^ or the aforementioned SMILES format.^29,30^ As explained in the Introduction, for the scaffolds, the structure generation is cumbersome because it usually represents a nontrivial structure that the user attempts to functionalize. Examples are inorganic semiconductor nanocrystals (as is the focus of our current work), but similar considerations hold for proteins, enzymes, dyes, and metal–organic frameworks*.* For this reason, one generally has the preconstructed scaffold (also called the core in the following when the method is applied to semiconductor nanocrystals) and passes its full structural information in the form of XYZ or PDB data. In the case of semiconductor nanocrystals, the builder NanoCrystal,^70^ which generates nanostructures with desired facets based on the Wulff construction method,^71^ can serve as a good starting point for generating initial scaffold structures.

Just as important as the actual structures is the definition of anchoring sites (*i.e.*, specific atoms) that determines how the scaffolds and ligands can be combined into a single structure. The specification of scaffold and ligand anchor atoms will be discussed in detail in their respective sections, including their relationship with the split and anchor options displayed in Figure S1.

### 2.2 Distribution of the Scaffold Anchoring Sites

Starting from a preprocessed scaffold (core), the core anchoring sites are user-specified dummy atoms that define where on the core’s surface the ligands can be attached. These dummy atoms are removed after the ligand is attached and can either be enumerated explicitly as a set of atomic indices or indicated by specific atomic symbols that should be considered anchoring sites.

**Figure 2:**
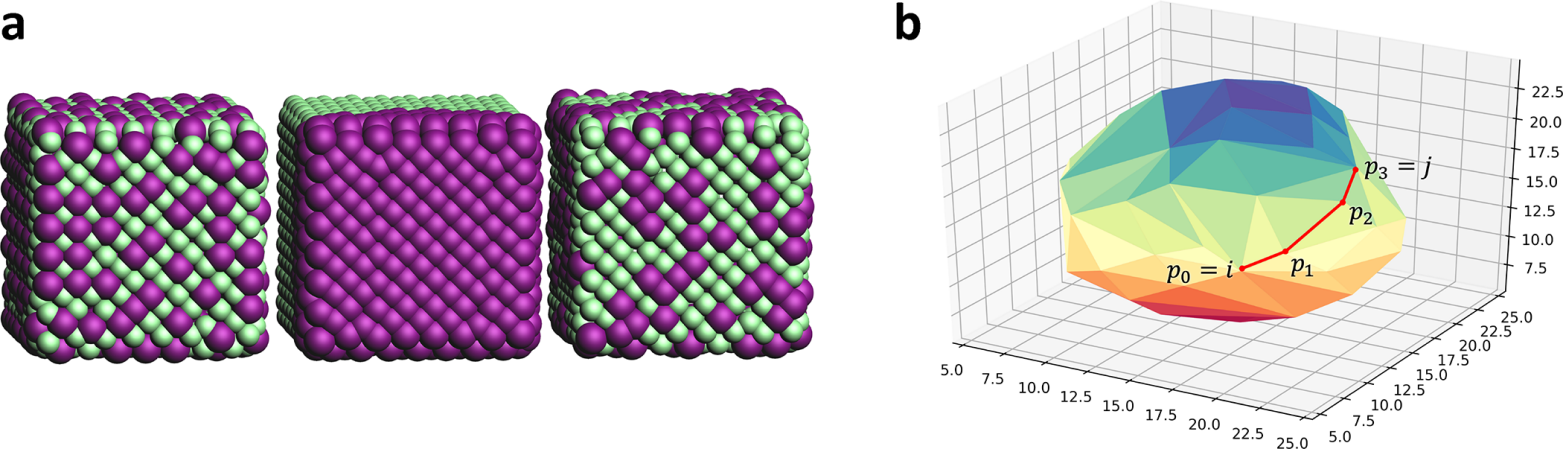
(a) Examples of a “uniform”, “cluster”, and “random” distribution of anchoring points (dummy atoms represented in purple color) on the surface that will be converted into organic ligands. The ligand surface coverage is set to 33%. The color code is provided to improve visual clarity between the various facets. The green color schematically indicates the atoms (whatever their type is) of the cubic lattice that will remain unchanged when dummy atoms are converted into organic ligands. (b) Core polyhedron representation. The vector **p** herein represents the shortest path between the atoms *i* and *j* along the surface.

The latter approach of treating all (dummy) atoms of a particular type as anchoring sites has the advantage of greatly simplifying the input, especially if one is interested in a full passivation of the scaffold surface at specific anchoring points. Whether such full passivation is appropriate for a particular scaffold depends on several factors, including the ligand size, the spacing between the anchoring sites on the scaffold, and the mutual attraction between the scaffold and the ligand. Due to above-mentioned caveats, a few schemes are available to automate the partial passivation of the scaffold (Figure 2a). This is especially important in cases where many ligands functionalize a scaffold, a typical case encountered in colloidal nanocrystals. For partial surface passivation, the full set of possible anchor atoms is reduced to a subset (of user-specified size) that can be distributed according to one or more of the following criteria:

- Uniform: Maximization of the (weighted) nearest-neighbor distance.
- Cluster: Minimization of the (weighted) nearest-neighbor distance.
- Random: Atoms are picked at random.

Note that one can freely combine the three distributions, allowing for a large degree of customization. For example, one could create a uniform distribution of *n*-sized clusters or a clustered/uniform distribution mixed with a degree of randomness (see Figure 2a).

As mentioned previously, the general idea behind the “uniform” and “cluster” distribution is, respectively, to maximize or minimize the nearest-neighbor distances. While exploring all possible combinations would in principle allow one to find a global optimum, such an approach will readily become prohibitively expensive as the number of combinations grows exponentially. Rather than attempting a full optimization, we therefore employ an iterative algorithm in which each to-be-passivated anchoring site is defined by maximizing/minimizing, respectively for “uniform”/“cluster” distributions, the weighted distance with respect to all previously picked anchoring sites (eq 1). As no previously picked anchor exists during the first iteration, the algorithm instead defaults to whichever anchor maximizes/minimizes the weighted distance with respect to all other anchoring atoms in the initial superset.
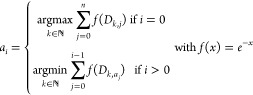
1

In eq 1, the variable  represents the distance matrix constructed from all-atom pairs in the initial superset of *n* atoms.  is a vector of unique atomic indices representing up to *n* atoms. For the “cluster” distribution, the argmax operation, as is used for *a*_*i* = 0_, is substituted for argmin, while argmax is used for all remaining elements. The default weighting factor, *f*(*x*) = *e*^–*x*^, ensures that nearest neighbors enter the equation with the largest weight.

By default, the distance matrix utilized in eq 1 represents the shortest paths between anchor–atom pairs through space. While this through-space definition is reasonable, especially with the increased weight of nearest neighbors, the distance between anchoring sites is in practice constrained by the scaffold surface. A more realistic representation of the distance criterion can therefore be desirable, *i.e.*, one based on the length of paths over the scaffold surface that connect these sites. Such a definition can be implemented by approximating the core as a polyhedron, constructed using SciPy’s implementation of the Qhull convex hull algorithm,^72^ and constraining all allowed paths to a graph defined by its edges. As is illustrated in Figure 2b, the result is a distance matrix that represents the shortest path along a scaffold’s (approximate) surface, rather than the shortest path through space.

All the scaffold–ligand structures obtained in this way can be used later on (after attachment of the ligands) as a starting point for geometry optimizations to verify which of the distribution is more stable, as well as employed as initial frames for molecular dynamic simulations, for example, for the replica exchange type of runs.

### 2.3 Ligand Anchors: Functional Group Recognition

Like their counterparts in the core, ligand anchors are defined by sites that are to be attached to the core’s anchors. In contrast to the definition of the core anchors, which require the use of dummy atoms, the ligand anchoring sites are located within functional groups and can thus readily be identified. Once a functional group (*e.g,*, a carboxylate) and an anchoring site within the aforementioned group (*i.e.*, the oxyanion) are defined, all the required information is available for the automatic identification (by searching for a characteristic pattern of atoms and bonds) of ligand anchoring sites for a given ligand specified in the input (see the “optional/ligand/anchor” option in Figure S1).

**Figure 3:**
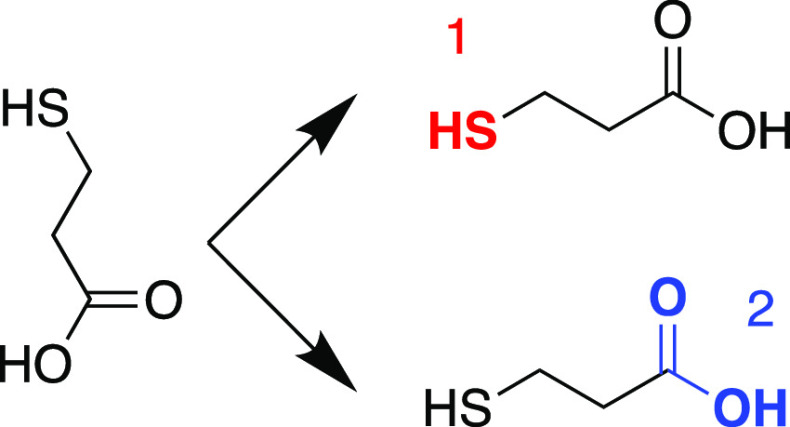
Example of automated functional group discovery in mercaptopropionic acid.

The specification of functional groups is done using SMILES strings,^29,30^ which provides a compact representation of organic systems. The user inputs desired functional groups in SMILES format to be anchored to the scaffold. Then, the user-provided ligand database is screened (by matching connectivity patterns with the aid of the RDKit library^29,32^) to select those ligands that contain the specified functional groups. These candidate ligands are henceforth referred to as “proto-ligands” and contain at least one of the desired functional groups and possibly more. A single copy is subsequently created of a proto-ligand for each valid functional group that it contains. These unique combinations of proto-ligand and functional group define a “ligand”, which is passed further along the workflow. For example, if we look for thiol and carboxylic acid functional groups, mercaptopropionic acid as a proto-ligand will produce two proper ligands, each with a different anchoring site (Figure 3). Depending on the user input, the functional groups can be used as anchors without modification or be deprotonated beforehand, *e.g.*, turning them into thiolate or carboxylate anions in the mercaptopropionic acid example (see the “optional/ligand/split” option in Figure S1).

### 2.4 Ligand Anchors: Biased Ligand Conformer Optimization

**Figure 4:**
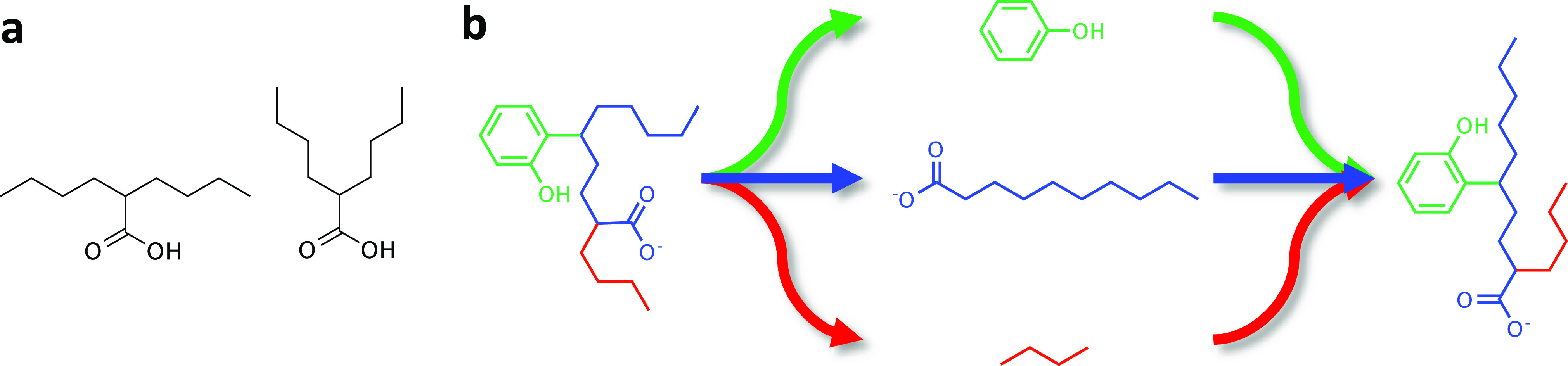
(a) Example conformations of a branched decanoic acid. (b) Example of a biased ligand conformation optimization where each branching group is separately linearized and then put together in the most linear configuration.

While SMILES strings contain all ingredients for constructing a particular molecular configuration, they do not contain information about the optimal three-dimensional conformation. In practice, this problem can generally be alleviated by using one of the numerous available tools for finding/approaching the global energy minimum of a ligand, for example, with the versatile GFN-xTB tight-binding methods.^73,74^ A challenge particular to the passivation of scaffolds with many anchoring sites is, however, that one does not deal with a single isolated ligand. This may render a minimum energy structure less suitable for use in ligand–scaffold combinations due to unfavorable ligand–ligand and ligand–scaffold interactions. A ligand conformational search must therefore somehow account for the expected interactions with the neighboring ligands and the scaffold. This is illustrated in Figure 4a, where the ligand’s particular global minimum (left) will result in severe interligand steric interactions once placed upon the scaffold’s surface.

There are therefore two main goals when deciding upon a suitable ligand conformation. The first one is that the resulting scaffold–ligand structures are stable even when all ligands have passivated the scaffold, *i.e.*, one should not employ a ligand conformation that will tear the system apart *via* repulsive interligand steric interactions. The second goal is that the conformational search is fast, also for large ligands. To deal with these challenges, a custom conformational search algorithm has been implemented (Figure 4b), which biases toward the creation of linear conformations that have the advantage of minimizing interligand steric interactions and prevent the collapse of the ligand on the scaffold. This aspect is particularly important for colloidal nanocrystals or systems alike, where ligands densely pack the scaffold surface and usually tend to elongate to minimize interligand steric repulsion. While not necessarily a global minimum, such elongated structures do typically yield low-energy conformations for the ligand–nanocrystal structure.

Our algorithm to produce linear conformers consists of the following six steps:

1. The (potentially) branched ligand is fragmented into linear components and capped with hydrogen atoms. Fragments are constructed such that the anchor atom is in the largest fragment.
2. The individual fragments are put in *anti*-periplanar conformations (Figure 4b). An exception is made whenever the anchor atom is part of a dihedral-defining bond, in which case, a *syn*-periplanar conformation is adopted.
3. The fragments are recombined one by one, removing the capping atoms and reforming the previously broken bonds. A set of three candidate conformers is generated by rotating the newly reformed bond, with a subsequent geometry optimization using an empirical universal force field (UFF)^75^ to ensure that each conformer corresponds to a stationary point.
4. The (weighted) perpendicular distance of each atom with respect to a central ligand vector is evaluated (eq 3 below), which is used as a measure for the “linearity” or the complementary “bulkiness” character of a conformation. The conformation that minimizes this weighted distance is optimized without further constraints and passed onto the next step. The definition of the ligand vector will be discussed in more detail in the next section.
5. Steps 3 and 4 are repeated until all fragments are reattached.
6. In cases where higher quality geometries are desired, the conformational search can be augmented with a final geometry optimization at a higher level of theory by interfacing CAT with an external engine. For example, one could optimize the ligand with a CHARMM-based force field^76−79^ using parameters automatically generated by MATCH,^80^ or if even greater accuracy is desired, one could choose a tight binding (*e.g.*, GFNn-xTB)^73,74^ or density functional calculation.^81,82^ All these external engines can be used if their executables are already pre-installed and, when required, licensed.

### 2.5 Ligand/Core Attachment

The general concept behind the scaffold–ligand passivation is similar to its previously described counterpart for organic systems:^64^ vectors and anchoring sites are defined for the scaffold and ligand, which are then used for rotating and translating the ligand(s) to their final position. A key difference between various scaffold types is, however, the presence or absence of covalent bonds with the organic ligands. Covalent bonds indeed play a key role in defining vectors for the cores. In the case of ionic inorganic nanocrystal scaffolds, where covalent bonds with the ligands are absent, the surface is generally defined by well-defined crystal facets; thus, the vectors of the inorganic core can be defined as those perpendicular to such facets. To identify the surface, CAT utilizes an idealized convex hull constructed from all scaffold anchors.^72^ The advantage of this approach is a guarantee of the surface’s convexity, the latter ensuring that all inorganic core vectors are either parallel or diverging. The assumption herein is that the surface can be reasonably represented by a convex hull, which might not hold for strongly concave surfaces or more exotically shaped cores (*e.g.* a torus). In such situations, the resulting regions of varying concavity give rise to converging inorganic core vectors, the latter being detrimental to interligand steric interactions once the surface is passivated with ligands.

On the other hand, for the vast majority of organic scaffolds where the interaction with the ligands is covalent, the procedure to define appropriate scaffold vectors is generally redundant. In that case, the presence of pre-existing bonds allows for easy identification of a scaffold vector, more specifically as the bond between the scaffold anchor and its direct neighbor. This simpler approach was already used by some of us in the construction of functionalized diimides for dye-sensitized photo-electrochemical cells.^64^

The ligand (unit-)vector  is determined by minimizing the (weighted) perpendicular distance (eq 2) of all atoms in the ligand. The origin is herein defined by its functional group-specific anchor atom, **r**_*i*_, representing a vector pointing from the anchor atom to atom *i* (eq 3).
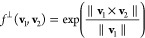
2
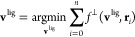
3

**Figure 5:**
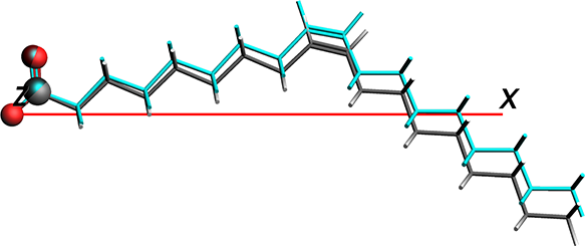
Difference in ligand rotation before (teal-colored oleate; eq 3) and after (gray-colored oleate) optimizing the ligand vector. **v**^lig^ is defined by the red horizontal line in both cases.

Note that as **v**^lig^ is both a parameter and a result, a guess is thus required for its initial value, which is then iterated until self-consistency is achieved. This initial trial vector is herein defined as the vector pointing from the ligand anchor atom to the mean position of all ligand atoms. The difference between the optimized and initial trial vectors is shown in Figure 5, which illustrates the already high quality of the trial vector as compared to the optimized vector.

After all vectors have been defined, the scaffold and the ligand(s) are combined into a single structure by aligning the vectors with each other. This is done in a sequential manner with each ligand being rotated along its vector’s axis such that the distance with respect to all previously defined neighboring ligands is maximized, thus minimizing steric interactions. A warning will be issued if any atoms between ligands are closer than 1.4 Å, indicating that further geometry optimizations are desirable. Ultimately, one obtains one or more ligand-passivated scaffolds. This procedure is identical for all scaffolds, be it a nanocrystal, a metal–organic framework, or an organic dye, with differences only in the definition of the scaffold vector as explained above.

### 2.6 Postprocessing

After assembling the ligand-passivated scaffolds, several optional workflows are available in CAT, ranging from a simple (constrained) geometry optimization to a workflow for computing ligand dissociation energies with the help of external engines such as AMS. At the end of each calculation, the resulting structures are exported to a user-specified format (*e.g.* XYZ or PDB), which can then be used for further calculations.

### 2.7 Examples

To demonstrate the efficiency of our tools for the preparation of realistic composite models and their transferability to a variety of different scaffold/ligand combinations, we demonstrate below the use of CAT for (i) the passivation of a nanocrystal surface by organic surfactants and (ii) the functionalization of a metal–organic framework (MOF) cavity with amino acids.

#### 2.7.1 Example 1

**Figure 6:**
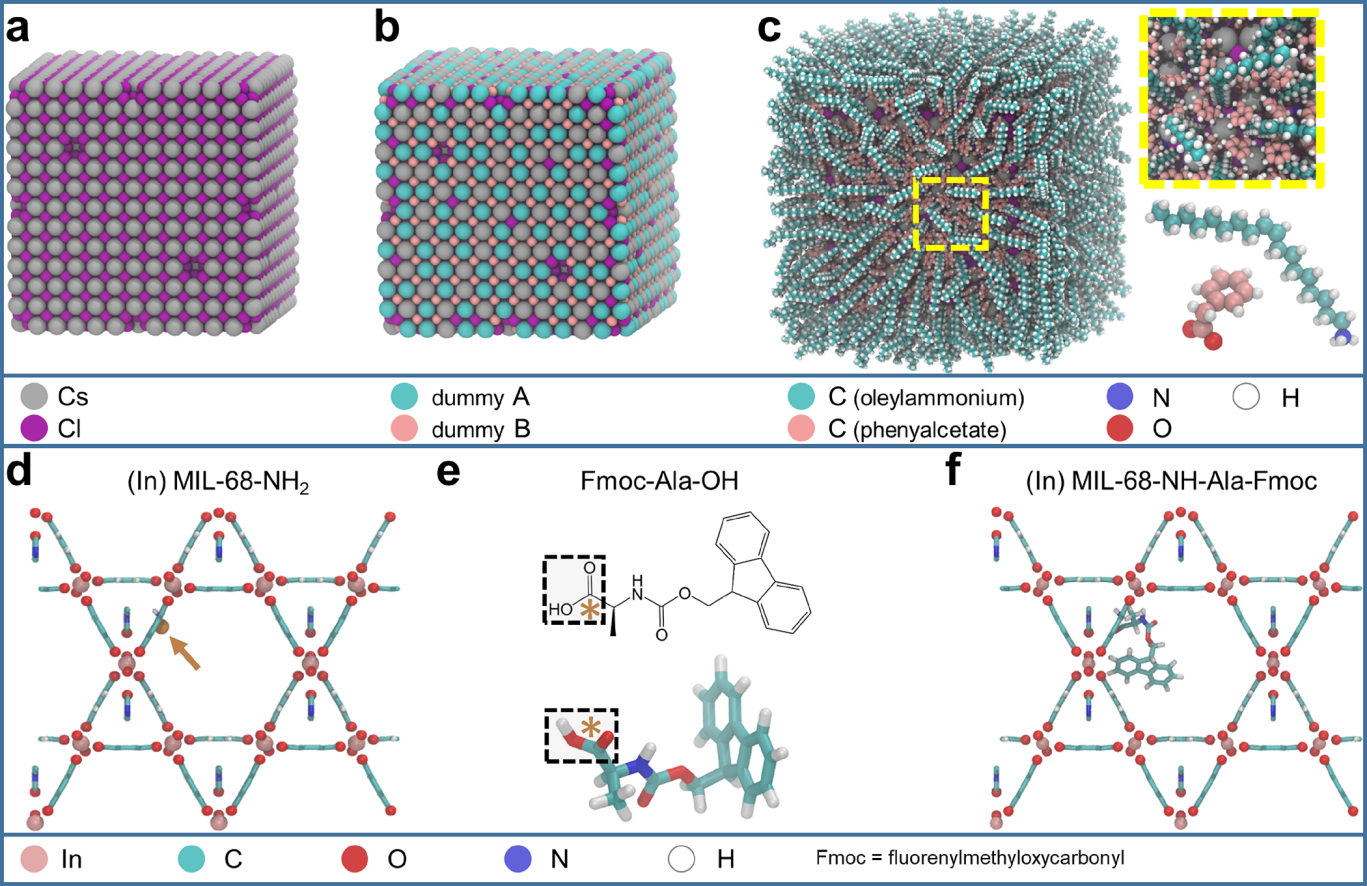
Top panel: Cs_2_AgInCl_6_ cubic nanocrystal model (a) enclosed by Cs-Cl-terminated facets, (b) after uniform replacement of 54% of the surface Cs by dummy atom A and 82% of the surface Cl by dummy atom B, and (c) after attachment of oleylammonium and phenylacetate ligands. The inset provides a closer look at the nanocrystal surface. The two ligands are also depicted alone to highlight the color code. Bottom panel: (d) (In) MIL-68-NH_2_ metal–organic framework model with the anchor site indicated by the arrow, (e) 2D (top) and 3D (bottom) representation of the Fmoc-Ala-OH ligand with the anchor site evidenced by a star, and (f) final (In) MIL-68-NH-Ala-Fmoc compound.

We start by illustrating the construction of a Cs_2_AgInCl_6_ double perovskite nanocrystal capped with both (cationic) oleylammonium and (anionic) phenylacetate ligands. This system is meant as a computational model for the Bi-doped Cs_2_Ag_1–*x*_Na_*x*_InCl_6_ nanocrystals synthesized by Zhang *et al.*(83) and characterized, according to nuclear magnetic resonance analysis, by a mixed oleylammonium/phenylacetate ligand shell (occupying respectively 54% of surface Cs sites/82% of surface Cl sites). In the first step, we built a cubic double perovskite nanocrystal model of about 6.0 nm in side by cutting a cubic Cs_2_AgInCl_6_ bulk system along the (100) planes, leaving Cs and Cl on the surface. As-cut, this nanostructure presents a stoichiometry of Cs_2197_Ag_864_In_864_Cl_5616_ corresponding to an excess of positive charge when each ion is considered in its more stable electronic configuration (*i.e.*, Cs^+^, Ag^+^, In^3+^, and Cl^–^). Consistent with previous works,^84,85^ we compensated this excess by removing 37 Cs ions from the surface, leading to a charge balanced Cs_2160_Ag_864_In_864_Cl_5616_ stoichiometry (Figure 6a). In a second step, we prepared the preprocessed nanocrystal core to carry out the partial surface passivation, *i.e.*, to define the positions where the cationic and anionic ligands should be attached. More specifically, we substituted 54% of the Cs surface atoms by dummy atom A and 82% of the Cl surface atoms by dummy atom B in a uniform manner (Figure 6b) using the dedicated CAT recipe: replace_surface (see the Python script in the Supporting Information, Figure S3). We then prepared the YAML input for CAT (Figure S4) by providing the above-mentioned inorganic core, covered by dummy atoms A and B, in an XYZ format. Here, the structure of the oleylammonium ligands is expressed as a SMILES string and replaces dummy atoms of type A. Moreover, the optional multi_ligand section is added and allows one to include the phenylacetate ligands, also expressed in the SMILES format, that replace dummy atoms of type B. Finally, we performed the multiligand attachment procedure by running the CAT workflow (YAML script executed in about 61 s on a single CPU), resulting in the double perovskite nanocrystal model passivated by a two-component ligand shell (Figure 6c), in line with the composition of the NCs synthesized in the experiments.^83^ This model was successfully employed as an initial structure to run classical MD simulations.^83^

#### 2.7.2 Example 2

In the second example, we aim to attach the protected enantiopure d-alanine, denoted as Fmoc-Ala-OH, to the (In) MIL-68-NH_2_ metal–organic framework to simulate the complex (In) MIL-68-NH-Ala-Fmoc compound obtained experimentally by Aguado *et al.*(86)*via* covalent postmodification (condensation). Following the guidelines provided by the computational study of Todorova *et al.*,^87^ we initially generated the metal–organic framework model from the isostructural (In) MIL-68^88^ by recasting it to its primitive cell (thereby facilitating the further grafting of ligands into a single hexagonal channel) before introducing an amino group to each benzene dicarboxylate linker. Here, we then identified one target NH_2_ group for the attachment procedure and labeled the H that will serve as the anchor site with dummy atom C (Figure 6d). As in the previous case, we inserted the preprocessed scaffold, its anchoring atom, and the SMILES string representing the Fmoc-Ala-OH ligand into the YAML input file (Figure S5). To account for the concave nature of the hosting pores in metal–organic frameworks, opposed to the convex surface of nanocrystals, we additionally invert the core vectors by setting their alignment to sphere_invert in the optional input section. Finally, to deal with the condensation reaction, we add more specialized information about the ligand anchor site to its input section: this includes the SMILES string of the functional group (carboxylic group here) as well as the indices of the atoms to be anchored (C) and removed (−OH) (Figure 6e).

By providing all these settings to CAT software (YAML script executed in about half a second on a single CPU), we finally obtained a (In) MIL-68-NH-Ala-Fmoc model (Figure 6f). This model also proves to be a suitable initial structure for further ab initio calculations, for example, for geometry optimizations using density functional theory.

## 3 Conclusions

In this work, we have developed a Python library named Compound Attachment Tool (CAT) for the automatic construction of composite chemical compounds, with an emphasis on inorganic nanocrystals passivated with organic ligands, although the library can be used for any type of system. The workflow consists of four distinct steps: the first step consists in defining the scaffold anchors by a user-provided set of dummy atoms. In the second step, complementary ligand anchor sites are identified by SMILES-based functional group recognition, allowing for parsing and filtering of a large set of (potential) ligands. In the third step, the ligand conformation is optimized to minimize interligand steric interactions when multiple ligands are present. This is accomplished by favoring *anti*-periplanar conformations and identifying (relatively) compact orientations of side chains. In the fourth and final step, the ligands are combined with the scaffold using the previously defined scaffold/ligand anchoring sites as attachment sites. The ligands are herein placed perpendicular with respect to the scaffold surface, and each ligand is furthermore rotated to maximize the distance with respect to neighboring ligands, thus minimizing interligand steric interactions. The resulting structure is the ligand-passivated (organic, inorganic, or hybrid organic–inorganic) scaffold. In addition to construction of the composite structural models themselves, various postprocessing workflows are available, *e.g.*, ligand dissociation energy calculations.

## Software and Data Availability

The CAT 1.0.0 source code is available on GitHub (https://github.com/nlesc-nano/CAT/tree/1.0.0), while binary distributions can be found on PyPi (https://pypi.org/project/nlesc-CAT/1.0.0/). Both are available under the GNU Lesser General Public License (version 3). All data referenced in Section 2.7 was generated using CAT 1.0.0, with the respective input and output files included in the Supporting Information.

## Supporting Information Available

Example YAML input files and illustrations of partially passivated nanocrystals (PDF)
Input file for the Cs_2_AgInCl_6_ double perovskite nanocrystal model (TXT)
YAML input file for the Cs_2_AgInCl_6_ double perovskite nanocrystal model (XYZ)
Output file for Cs_2_AgInCl_6_ double perovskite nanocrystal model (XYZ)
Input file for (In) MIL-68-NH_2_ (TXT)
YAML input file for (In) MIL-68-NH_2_ (XYZ)
Output file for (In) MIL-68-NH_2_ (XYZ)
README file (TXT)

## Supplementary Material

ci2c00690_si_002.txt

ci2c00690_si_003.xyz

ci2c00690_si_004.xyz

ci2c00690_si_005.txt

ci2c00690_si_006.xyz

ci2c00690_si_007.xyz

## Abbreviations

CAT: Compound Attachment Tool
ASE: Atomic Simulation Environment
VASP: Vienna Ab initio Simulation Package
ADF: Amsterdam Density Functional program
NOMAD: Novel Materials Discovery
GDB-13: Generated Database with up to 13 atoms
SMILES: Simplified Molecular-Input Line-Entry System
SELFIES: Self-Referencing Embedded Strings
YAML: YAML Ain’t Markup Language
PLAMS: Python Library for Automating Molecular Simulation
PDB: Protein Databank format
InChI: International Chemical Identifier
GFN-xTB: Geometry Frequency Noncovalent Extended Tight Binding
MOF: metal–organic framework
PyPi: The Python Packaging Index
GNU: GNU’s Not Unix!
