# Supplementary material for: CAT: A Compound Attachment Tool for the Construction of Composite Chemical Compounds
Source: J Chem Inf Model. 2022 Oct 31;62(22):5525–35. doi: 10.1021/acs.jcim.2c00690 (PMC9976287; doi:10.1021/acs.jcim.2c00690)
Supplement: Supplementary file 1 — ci2c00690_si_001.pdf [file ci2c00690_si_001.pdf]

Supporting Information for:

# CAT: A Compound Attachment Tool for the construction of composite chemical compounds

*Bas van Beek[#], Juliette Zito,[†,ß] Lucas Visscher[*,#] and Ivan Infante[*,†,‡,¶]*

# Division of Theoretical Chemistry, Faculty of Science, Vrije Universiteit Amsterdam, de Boelelaan 1083, 1081 HV Amsterdam, the Netherlands

ß Dipartimento di Chimica e Chimica Industriale, Università degli Studi di Genova, Via Dodecaneso 31, 16146 Genova, Italy

† Department of Nanochemistry, Istituto Italiano di Tecnologia, Via Morego 30, 16163 Genova, Italy

‡ BCMaterials, Basque Center for Materials, Applications, and Nanostructures, UPV/EHU Science Park, Leioa 48940, Spain

¶ Ikerbasque Basque Foundation for Science Bilbao 48009, Spain

* ivan.infante@bcmaterials.net

```yaml
path:
.

input_cores:
   -  Cd68Se55.xyz

input_ligands:
   -  C(C=O)O
   -  CC(C=O)O
   -  CCC(C=O)O

optional:
   core:
       anchor: Cl
   ligands:
       anchor: O(C=O)[H]
       split: True
```

**Figure S1**: An example of YAML input file.

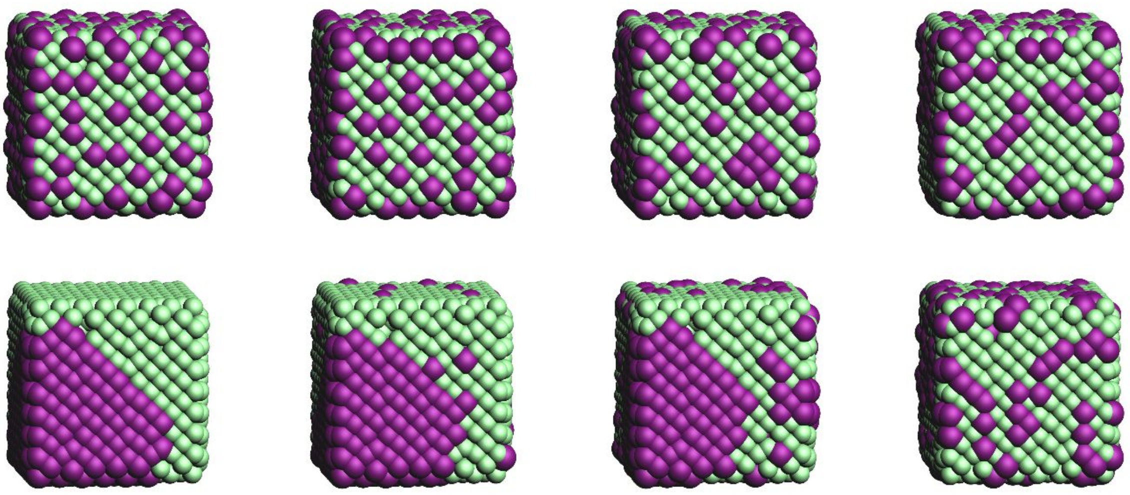

**Figure S2**: "uniform" (top) and "cluster" (bottom) distributions with varying degrees of randomness at a surface coverage of 25%. From left to right the degree randomness is increased from 0, 25%, 50% and 100%.

```python
from scm.plams import Molecule
from CAT.recipes import replace_surface

mol = Molecule('6nm_DP_model.xyz')

mol_tmp = replace_surface(
    mol, symbol='Cs', symbol_new='Rb',
    f=0.54, mode='uniform', displacement_factor=0.9,
)
mol_new = replace_surface(
    mol_tmp, symbol='Cl', symbol_new='Br',
    f=0.82, mode='uniform', displacement_factor=0.9,
)
mol_new.write('6nm_DP_model_RbBr.xyz')
```

**Figure S3**: Python script used to prepare a $Cs_2AgInCl_6$ double perovskite core for the partial surface passivation. The dedicated CAT recipe, *replace_surface*, is called to substitute 54% of the Cs surface atoms by dummy atom A (arbitrarily chosen to be 'Rb' here) and 82% of the Cl surface atoms by dummy atom B (arbitrarily chosen to be 'Br' here) in a uniform manner. Requires both CAT and its Nano-CAT sister package (`pip install nlesc-CAT nano-CAT`).

```yaml
path: null

input_cores:
    - 6nm_DP_model_RbBr.xyz:
        guess_bonds: False

input_ligands:
    - CCCCCCCC/C=C\CCCCCCCC[NH3+] # oleylammonium

optional:
    core:
        anchor: Rb  # dummy atom A

    ligand:
        split: False

    qd:
        multi_ligand:
            ligands:
                - C1=CC=C(C=C1)CC(=O)[O-] # phenylacetate
            anchor:
                - Br # dummy atom B
```

**Figure S4**: CAT input file for the capping of the $Cs_2AgInCl_6$ double perovskite core with both oleylammonium and phenylacetate using the multi-ligand attachment procedure.

```yaml
path: null

input_cores:
    - In-MIL-68-NH2_Cl.xyz

input_ligands:
    - CC(C(=O)O)NC(=O)OCC1C2=CC=CC=C2C3=CC=CC=C13 #Fmoc-Ala-OH

optional:
    core:
        anchor: Cl # dummy atom C
        allignment: surface_invert
    ligand:
        anchor:
            - group: "C(=O)O[H]"
              group_idx: 0
              remove: [2, 3]
```

**Figure S5**: CAT input file to graft the protected enantiopure D-alanine, Fmoc-Ala-OH, to the (In) MIL-68-$NH_2$ metal-organic framework marked by dummy atom C (arbitrarily chosen to be 'Cl' here).
